# Tissue calcinosis in dermatomyositis - potential role of combination therapy with JAK inhibitors. Case report and literature review

**DOI:** 10.3389/fimmu.2026.1903238

**Published:** 2026-09-16

**Authors:** Paulina Janiak, Żaneta Smoleńska, Anna Wardowska, Michał Chmielewski

**Affiliations:** Department of Rheumatology, Clinical Immunology, Geriatrics and Internal Medicine, Medical University of Gdańsk, Gdańsk, Poland

**Keywords:** calcinosis, calcinosis cutis, dermatomyositis, Janus kinase inhibitors, systemic sclerosis

## Abstract

Calcinosis is a disabling and difficult-to-treat complication of adult dermatomyositis (DM), juvenile dermatomyositis (JDM), and systemic sclerosis (SSc), for which no standardized therapy is currently available. Growing evidence implicating JAK/STAT signaling in dermatomyositis has prompted interest in Janus kinase inhibitors (JAKi) as a therapeutic option. We describe a 52-year-old woman with long-standing juvenile-onset dermatomyositis and extensive, treatment-refractory calcinosis who received off-label baricitinib after multiple previous therapies. After one month, the patient reported improved general well-being and no new skin ulcerations. During the four-month treatment course, pre-existing microcytic anemia worsened and required blood transfusion, prompting discontinuation of baricitinib because a treatment-related contribution could not be excluded. This case provides a hypothesis-generating observation of possible early symptomatic benefit during JAK inhibition while highlighting the uncertainty regarding efficacy and safety. Prospective studies using standardized clinical, functional, imaging, and safety outcomes are required.

## Introduction

Calcinosis is the accumulation of insoluble calcium salt crystals in the skin, muscles, tendons, and subcutaneous tissue. It is one of the manifestations of DM, JDM, and SSc, typically developing later in the disease course (1). It often negatively affects patients’ quality of life, causing impaired mobility, skin ulcerations, pain, and tenderness (2). Several potential mechanisms underlying calcinosis have been proposed. Chronic inflammation plays a key role in the formation of calcium deposits. However, in patients with SSc, calcinosis appears to be more closely associated with vascular ischemia and repetitive mechanical trauma. It commonly occurs in the distal part of the body, and the main mineral component is bone-like hydroxyapatite (3, 4). In contrast, the pathogenesis of calcinosis in DM and JDM is linked to mitochondrial intracellular calcification. It typically involves the muscles and subcutaneous tissue of the trunk and limbs, with carbonate apatite as the predominant mineral component (2, 5, 6). Calcinosis develops and progresses over time and is more frequently observed in patients with JDM and SSc compared to those with DM. Serum calcium and phosphatase levels are usually within normal ranges (2).

## Case report

A 52-year-old woman diagnosed with JDM at the age of 10 and complicated by calcinosis, presented to the rheumatology outpatient clinic for a routine follow-up. She reported chronic muscle weakness and myalgia. In addition, arthralgia, dry mouth and eyes, and fecal incontinence were noted. Her medical history included hypertension, hyperlipidemia, depression, osteoporosis, and recurrent microcytic anemia requiring intravenous iron supplementation. Physical examination revealed extensive soft-tissue calcinosis of the upper and lower extremities and the gluteal region; several lesions were ulcerated. Hypertrophy of the distal lower limbs was also noted (**Figure 1**). At baseline, hemoglobin (Hb) was 11.6 g/dL (reference range 12.0–15.0), creatine kinase (CK) was 245 U/L (29–168), and lactate dehydrogenase (LDH) was 227 U/L (125–220); hyperuricemia and hypertriglyceridemia were also present. Inflammatory markers, complement components C3 and C4, serum calcium, phosphorus, and vitamin D were within reference ranges. Immunologic testing was positive for anti-Mi-2β (+), anti-nuclear matrix protein 2 (anti-NXP2) (+++) antibodies, with borderline positivity for anti-polymyositis/scleroderma (anti-PM/Scl), and anti–alanyl-tRNA synthetase (anti-PL-12) antibodies. The Schirmer’s test was negative, confirming normal tear production, while the Saxon’s test was positive indicating oral dryness. Hand radiographs showed degenerative changes; a more detailed joint-level radiographic description was unavailable for retrospective review (**Figure 2A**), whereas radiographs of the lower limbs revealed calcinosis deposits in soft tissues (**Figures 2B, C**). Abdominal computed tomography (CT) was unremarkable; however, chest CT revealed calcinosis within the soft tissues of the chest and dorsal region, which was also observed on lumbar and pelvic radiographs (**Figure 3**). Due to lower-limb hypertrophy and suspected edema, ultrasonography (USG) of the lower extremity was performed, revealing overgrowth of adipose tissue.

**Figure 1:**
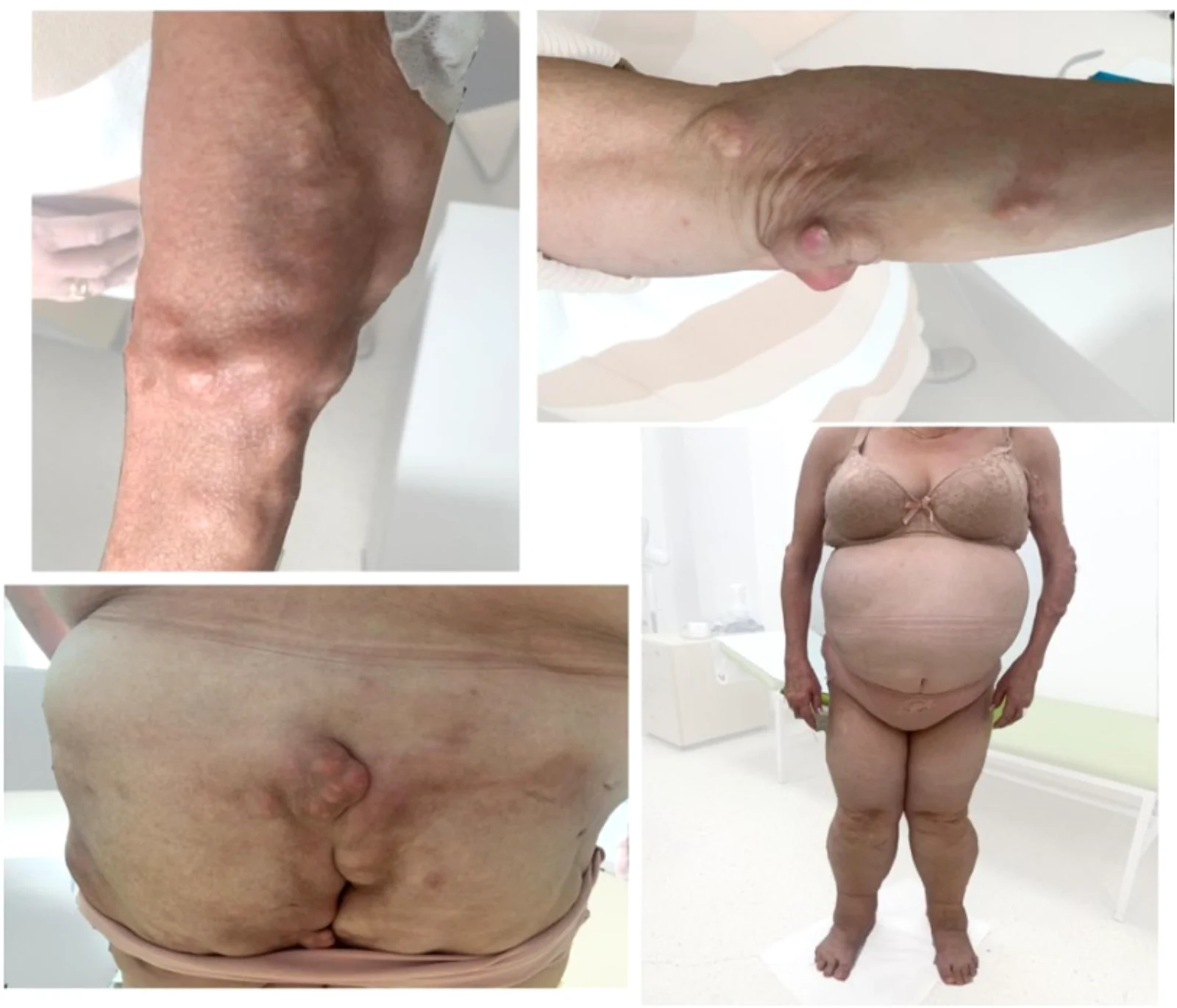
Clinical appearance of extensive soft-tissue calcinosis involving the extremities and gluteal region, including ulcerated lesions.

**Figure 2:**
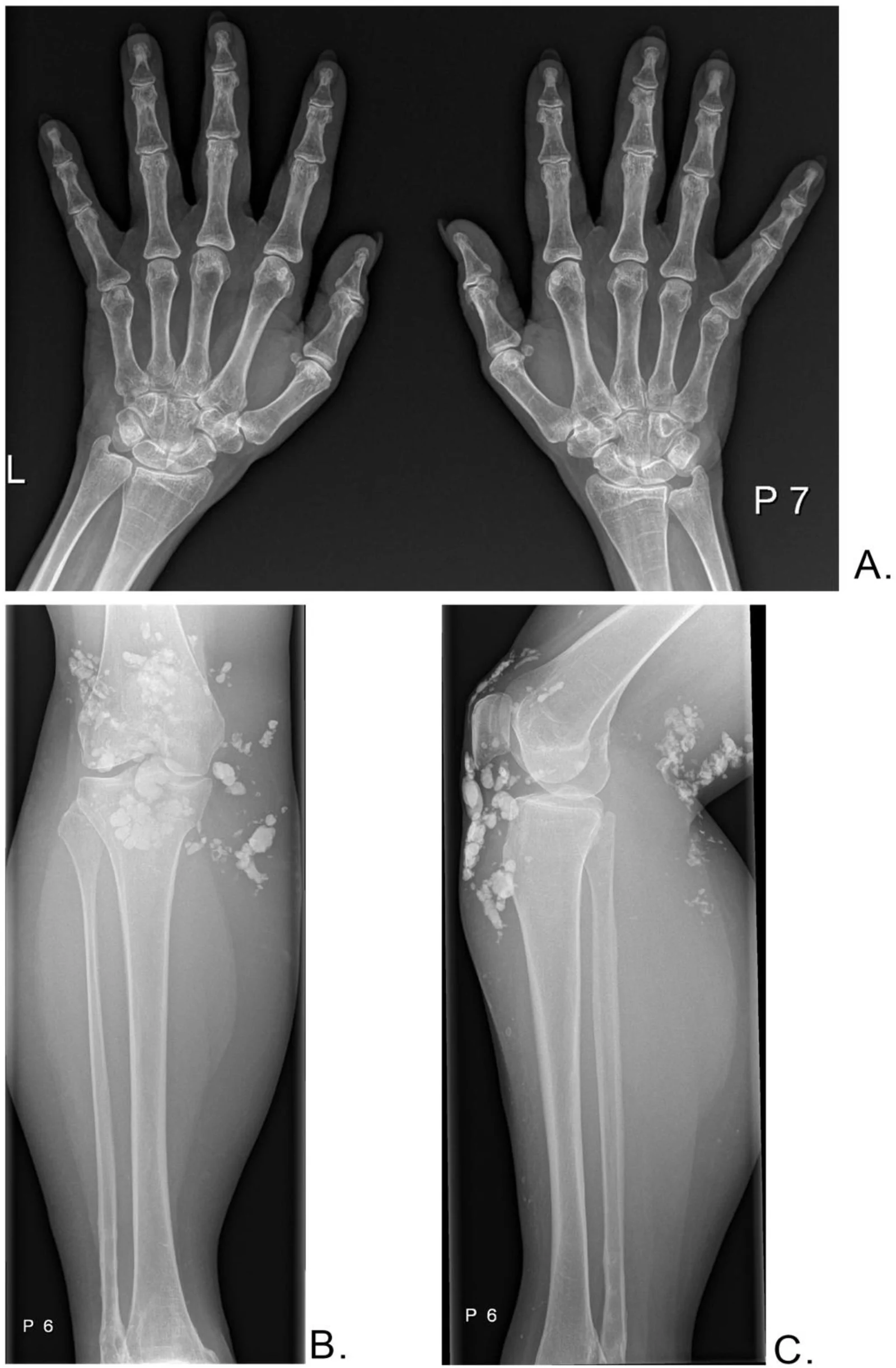
Hand radiographs revealed bilateral joint-space narrowing of the interphalangeal I (IP I) joints and the right first metacarpophalangeal (MCP I) joint, with subchondral sclerosis and marginal osteophyte formation. **(A)** and lower-limb radiographs showing extensive soft-tissue calcification **(B, C)**.

**Figure 3:**
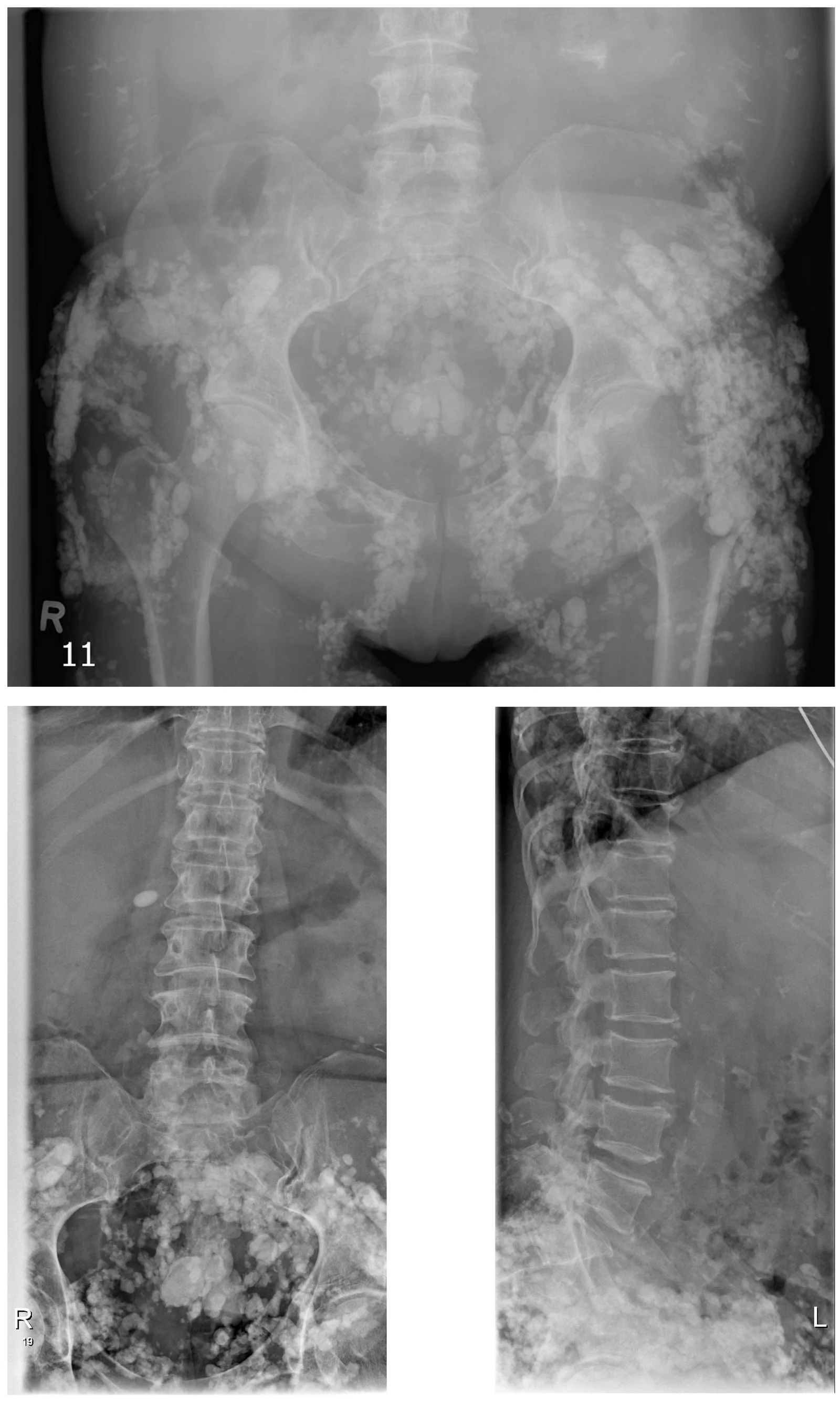
Radiographic evidence of extensive soft-tissue calcification in the lumbar and pelvic regions.

The historic diagnosis of JDM had been made at 10 years of age and the original diagnostic documentation was not available for retrospective reassessment. The diagnosis of JDM was supported by chronic muscle weakness and myalgia, mildly elevated CK and LDH, anti-NXP2 positivity, and longstanding calcinosis. Systemic sclerosis, mixed connective tissue disease, antisynthetase syndrome, and Sjögren disease were considered because of the clinical manifestations, positivity for anti-PM/Scl and anti-PL-12. However, Raynaud phenomenon, puffy fingers, sclerodactyly, mechanic’s hands, interstitial lung disease, anti-U1 ribonucleoprotein (anti-U1-RNP), and characteristic systemic-sclerosis antibodies were not documented. The patient had confirmed xerostomia, which was mostly likely treatment- related, particularly due to the use of psychiatric medications. Moreover, specific antibodies for Sjogren disease were not detected and there was no evidence of xerophthalmia. The principal diagnostic considerations are summarized in **Table 1**.

**Table 1:** Clinical characteristics, differential diagnostic considerations, and treatment course.

A. Clinical presentation and available investigations			
Clinical phenotype	Laboratory findings	Immunology	Imaging
•JDM diagnosed at age 10 (historical diagnosis) •chronic muscle weakness, myalgia, and arthralgia •extensive calcinosis with ulcerated lesions •distal lower-limb hypertrophy •xerostomia, xerophthalmia, and fecal incontinence	•Hb 11.6 g/dL (12.0-15.0) •CK 245 U/L (29-168) •LDH 227 U/L (125-220) •inflammatory markers not elevated •C3/C4 within reference ranges •calcium, phosphorus, and vitamin D within reference ranges	•anti-NXP2 (+++) •anti-Mi-2beta (+) •borderline anti-PM/Scl •borderline anti-PL-12	•radiographs and CT: extensive soft-tissue calcinosis of the limbs, chest/dorsal and lumbar/pelvic region •hand radiographs: degenerative changes •lower-limb USG: adipose-tissue overgrowth
B. Differential diagnostic considerations				
Diagnosis	Supporting or documented findings	Findings limiting or arguing against		
JDM / DM	juvenile onset, chronic muscle symptoms, elevated CK/LDH, anti-NXP2 and anti-Mi-2beta detected, longstanding calcinosis	original diagnostic documentation; standardized muscle-strength assessment, and characteristic juvenile-onset skin findings were unavailable		
Systemic sclerosis	arthralgia, calcinosis, gastrointestinal symptoms, borderline anti-PM/Scl.	no documented Raynaud phenomenon, puffy fingers, sclerodactyly, interstitial lung disease or characteristic systemic-sclerosis antibodies		
Mixed connective tissue disease	myalgia, arthralgia, xerostomia and xerophthalmia	anti-U1-RNP, Raynaud phenomenon, puffy fingers were not documented; negative Schirmer’s test		
Antisynthetase syndrome	myalgia, arthralgia, borderline anti-PL-12	anti-PL-12 was borderline; no documented Raynaud phenomenon, mechanic's hands, interstitial lung disease.		
Sjögren disease	xerostomia, xerophthalmia	negative Schirmer’s test, specific antibodies not detected		
C. Treatment and follow-up timeline				
Before baricitinib	Treatment initiation	After 1 month	At 4 months and follow-up	
•recurrent microcytic anemia had previously required intravenous iron •long-term glucocorticoids, MTX, diltiazem, bisphosphonates, vitamin D, IVIG	•baricitinib 4 mg once daily (off-label) •prednisone 10 mg/day, diltiazem 60 mg morning and 120 mg evening, zoledronic acid	•no new skin ulcerations •improved general well-being •CK and LDH did not decrease •inflammatory markers remained unchanged.	•anemia worsened and required transfusion •baricitinib discontinued after 4 months •Hb remained low but stable, calcinosis progressed (at 1 year after baricitinib discontinuation)	

The patient’s treatment records for JDM included long-term glucocorticosteroid therapy later combined with methotrexate (MTX) (15–25 mg/week), diltiazem (60 mg in the morning and 120 mg in the evening), monthly ibandronic acid which was subsequently switched to annual zoledronic acid, daily vitamin D supplementation. The medical history also included several sessions of intravenous immunoglobulin (IVIG) administered at a dose of 2 g/per kg of body weight; however, this treatment was considered ineffective. The patient continued to experience disease-related symptoms, including painful, ulcerative skin nodules and muscle weakness, which significantly affected her daily functioning. Baricitinib (4 mg once daily) was therefore introduced as an experimental off-label treatment after informed consent. The therapy was combined with prednisone 10 mg once daily, diltiazem 60 mg in the morning and 120 mg in the evening, and zoledronic acid. After one month, the patient reported no new skin ulcerations and an overall improvement in well-being; CK and LDH did not decrease, and inflammatory markers remained unchanged. During treatment, anemia progressed and eventually required blood transfusion. Because a treatment-related contribution could not be excluded, baricitinib was discontinued after four months. An extensive evaluation, including hematological and gastroenterological assessments, was subsequently performed. The persistent anemia was considered to be associated with the underlying JDM, while concomitant iron deficiency was attributed to gastritis caused by chronic *Helicobacter pylori* infection. Baricitinib was not reintroduced, and no more selective JAK inhibitor was initiated because of the limited availability of alternative agents. One year after discontinuation of baricitinib, hemoglobin remained below the reference range but was stable, while calcinosis continued to progress.

## Discussion

Calcinosis is a significant clinical problem in patients with DM, JDM, and SSc. Significant differences in the pattern and characteristics of calcinosis are observed between DM and SSc (**Table 2**). The prevalence of calcinosis is estimated at approximately 20% in DM and 20-40% in JDM (7, 8). Whereas, in SSc patients, it ranges from 18% to 49%. In SSc, calcinosis typically develops approximately 10 years after the first non-Raynaud’s syndrome. In contrast, in JDM, it usually appears within 2–3 years after disease onset, particularly in cases of persistent disease activity and suboptimal disease control. In DM, calcinosis represents a manifestation of cutaneous involvement, with the risk increasing over time (2, 9, 10). Calcinosis in DM, JDM, and SSc is associated with the presence of disease -specific autoantibodies. Valenzuela et al. reported that anti-MDA-5 and anti-Ro-52 antibodies were significantly more frequent in patients with DM and calcinosis compared with those without calcinosis (35.7% vs. 9.8%, p = 0.006, and 42.9% vs. 17.9%, p = 0.02, respectively). Anti-NXP-2 antibodies were also more frequently detected in patients with calcinosis (28.6% vs. 10.7%, p = 0.06) (11). Similarly, Albayda et al. demonstrated that calcinosis occurred more frequently in patients positive for anti-NXP-2 antibodies (30% vs. 17%; p = 0.02) (12), and Wang et al. confirmed that the presence of anti-NXP-2 is associated with an increased risk of calcinosis development (13). This association has been confirmed in JDM cohorts (14, 15). Tansley et al. reported that 43% of anti-NXP-2 positive JDM patients developed calcinosis, compared to a lower proportion among those without this antibody (15). In patients with SSc, anti-centromere antibodies (ACA), RNA-polymerase-III (RNAP3), and anti-topoisomerase (anti-Scl-70) antibodies are more frequently observed (2, 4, 16). Additionally, an association between anti-PM/Scl and the development of calcinosis has been reported in both SSc and DM/JDM (17, 18).

**Table 2:** Comparison of calcinosis in DM/JDM and SSc.

Feature	DM/JDM	SSc
Prevalence	approximately 20% in adult DM, 20-40% in JDM	approximately 18-49%
Mechanisms of calcification	mitochondrial dysfunction, intracellular mitochondrial calcification	vascular ischemia, tissue hypoxia, repetitive mechanical trauma
Mineral component	carbonate apatite	bone-like hydroxyapatite
Typical distribution	subcutaneous tissue and muscles of the trunk and limbs	distal extremities, pressure or trauma-prone sites
Characteristic antibodies	anti-NXP2, anti-MDA5, anti-Ro52, and anti-PM/Scl	anti-centromere antibodies (ACAs), anti-RNA polymerase III, anti-Scl-70, anti-PM/Scl

Although the precise pathogenesis of calcinosis remains incompletely understood, it is currently regarded as a multifactorial process involving chronic inflammation, mitochondrial dysfunction, vascular hypoxia, and repetitive microtrauma. Pro-inflammatory cytokines, including IL-1β, IL-6, TNF-α, and INF-I play a central role in disease progression. In SSc, hypoxia-related mechanisms appear to be particularly relevant. Markers of oxidative stress, such as glucose transporter-1 (GLUT-1) and vascular endothelial growth factor (VEGF), are upregulated. VEGF, a potent angiogenic factor, may contribute to abnormal osteoclast activity and increased bone resorption (19, 20). Davies et al. reported increased expression of bone matrix proteins involved in ectopic calcification (19). Distinct pathogenic pathways have been described in JDM and DM. Davuluri et al. identified mitochondrial calcification as a key mechanism underlying tissue calcinosis (20). Multiple triggers, including cell death, trauma, hypoxia, and excessive mitochondrial reactive oxygen species (mtROS), promote the accumulation of calcium and phosphate within mitochondria. Intramitochondrial calcification amplifies inflammation, leading to mitochondrial damage and subsequent extracellular deposition of carbonate apatite. Although osteoclasts and macrophages attempt to clear these deposits, this process appears to be impaired in JDM and DM (21, 22).

Currently, there are no standardized treatment guidelines for calcinosis. Commonly used therapies include bisphosphonates, which inhibit osteoclast activity and reduce pro-inflammatory cytokine production; calcium channel blockers, which decrease intracellular calcium influx; and sodium thiosulfate, which may promote calcium solubilization and improve local vasodilation. Given the central role of chronic inflammation, anti-inflammatory therapies have been explored, such as a rational approach to limit disease progression (5, 22). Biologic agents have been increasingly used in selected cases. Rituximab, a chimeric anti-CD20 monoclonal antibody, has shown potential benefits, particularly in patients with pulmonary involvement. TNF inhibitors, such as infliximab or adalimumab, may reduce TNF levels, which are elevated in patients with calcinosis. Intravenous immunoglobulins (IVIG) have also been reported to be beneficial, particularly in DM, possibly through modulation of T- cell activity, although the exact mechanism remains unclear (1, 21–23).

Based on emerging evidence, Janus inhibitors have gained attention as a potential therapeutic option in DM and JDM-associated calcinosis. Janus kinases (JAK1, JAK2, JAK3, TYK) are receptor-linked tyrosine kinases that activate the signal transducer and activator of transcription protein (STAT). The JAK/STAT pathway regulates gene expression in response to cytokines, growth factors, and hormones, and plays a critical role in immune homeostasis. However, its dysregulation may lead to excessive immune activation and autoimmunity. In DM and JDM, the JAK/STAT pathway is frequently overactivated (24, 25). Type I interferons are thought to play a role in this process. Increased IFN signaling in muscle, skin, and serum promotes the expression of interferon-regulated genes via the JAK/STAT pathway, leading to sustained cytokine production. Therefore, inhibition of this pathway represents a promising strategy to suppress inflammation and downstream pathogenic processes. Moreover, dysregulation of JAK/STAT signaling may contribute to mitochondrial dysfunction and disturbed cellular calcium homeostasis, further promoting calcinosis (24–26).

Several JAKi are approved for the treatment of immune-mediated inflammatory diseases. These include tofacitinib, baricitinib, upadacitinib, fligotinib, and peficitinib, which differ in their selectivity for individual JAK isoforms. Tofacitinib primarily inhibits JAK1 and JAK3 (with partial activity against JAK2), baricitinib targets JAK1 and JAK2 with similar affinity, peficitinib acts as a pan-JAKi, whereas upadacitinib and filgotinib are more selective for JAK1, potentially reducing off-target effects. Despite their established efficacy in diseases such as rheumatoid arthritis, psoriatic arthritis, and inflammatory bowel disease, JAKi have not yet been approved for the treatment of DM or JDM. Baricitinib has been shown to downregulate IFN-related gene expression (24–32). Papadopoulou et al. reported clinical improvement in patients with treatment-resistant JDM receiving baricitinib, and several studies have demonstrated its efficacy and safety in refractory DM and JDM (28–32). Tofacitinib has also been reported to improve skin lesions and muscle strength, while brepocitinib, a TYK2/JAK1 inhibitor, is currently under clinical investigation in DM, with promising preliminary results (32–34) (**Table 3**).

**Table 3:** Published reports of JAK inhibitor treatment in DM/JDM-associated calcinosis.

Study / population	Calcinosis phenotype	JAK inhibitor	Prior or concomitant therapy	Calcinosis-specific outcome	Follow-up / assessment	Adverse events
Wendel et al., 2019; 54-year-old woman (35)	hip, hand, buccal, and gluteal deposits, ulceration	tofacitinib	prednisone, MTX, and multiple anti-calcinosis agents	ulcers resolved, deposits stable or regressive	28 weeks/ clinical and imaging assessment	NR
Wendel et al., 2019; 55-year-old woman (35)	DM/ILD, gluteal and elbow deposits	tofacitinib	multiple immunosuppressive agents	regression reported, no new deposits	NR/ clinical and imaging assessment	weight gain
Shneyderman et al., 2021; 3 adult women (33)	multifocal calcinosis	tofacitinib	NR	reduced lesion size or regression, pain improvement	3 months/ clinical and imaging assessment	NR
Paik et al., 2021; 10 adults (36),	refractory DM, 3 calcinosis cases	tofacitinib	multiple immunotherapies	improvement in the small calcinosis subgroup	12 weeks/ NS	NS
Corbella-Bagot et al., 2024; 14 adults (37)	refractory DM, 2 calcinosis cases	tofacitinib or baricitinib	multiple immunotherapies	one case improved, one did not respond	NS	NS
Nowak et al., 2025; 31-year-old woman (38)	PsA/DM overlap; periarticular dystrophic calcinosis	upadacitinib	prednisolone, MTX, rituximab, MMF, minocycline, and pamidronate	radiographic resolution of periarticular calcifications	NS/radiographic assessment	NS
Papadopoulou et al., 2019; 11-year-old girl (28)	refractory JDM with pre-existing calcinosis	baricitinib	multiple immunotherapies and glucocorticoids	no new calcinosis reported, calcinosis regression not demonstrated	6 months/ clinical disease-activity and functional assessment	NR
Kim et al., 2021; 4 patients aged 5.8-20.7 years (29)	refractory JDM, 2 calcinosis cases	baricitinib	glucocorticoids and multiple immunotherapies	calcinosis specific outcome not reported	4 weeks/ muscle strength and global disease activity assessment	NR
Agud-Dios et al., 2022; 7-year-old child (30)	JDM associated calcinosis	baricitinib 2 mg/day	MTX, IVIG, glucocorticoids, bisphosphonate, physical therapy	deposits softened, objective change in calcific burden not reported	outcome at 5 months, 7-month safety follow-up/ clinical assessment	NR
Wang et al., 2025; 27 children (40)	refractory JDM, 3 calcinosis cases	baricitinib	multiple immunotherapies	calcinosis improvement reported	36 months/ retrospective cohort/NS	one VZV infection reported
Oshima et al., 2026; 10-year-old child (39)	extensive calcinosis	baricitinib 2 mg/day	multiple immunotherapies and bisphosphonate	no calcinosis progression	NS/ clinical and imaging assessment	NR

AE, adverse event; ILD, interstitial lung disease; MMF-mycophenolate mofetil; NR, not reported; NS, not specified; PsA, psoriatic arthritis; VZV, varicella-zoster virus.

Published evidence of JAK inhibition in calcinosis consists mainly of pediatric JDM reports and small adult DM series, with heterogeneous drugs, follow-up periods, and outcome measures (**Table 3**). The present case extends this limited experience to an adult with very long-standing juvenile-onset disease and provides a four-month treatment course together with one-year post-discontinuation follow-up. The early absence of new ulcerations and improved well-being, the subsequent hematological and gastroenterological evaluations, and later progression of calcinosis provide an additional real-world, hypothesis-generating observation that may help define questions for future studies of effectiveness, patient selection, durability, and safety. The available record includes exact baseline laboratory values, the four-month treatment duration, a subsequent hematological and gastroenterological assessments, and one-year post-discontinuation follow-up. The early clinical observations therefore provide a relevant hypothesis-generating signal, although the effect on calcific burden could not be objectively quantified. The contribution of baricitinib to anemia remains uncertain because anemia pre-dated treatment and the later evaluation identified underlying-disease and iron-deficiency-related factors.

## Conclusion

In this patient with long-standing juvenile-onset dermatomyositis and extensive refractory calcinosis, the absence of new skin ulcerations and improved well-being after baricitinib therapy suggest a possible early clinical benefit. The four-month treatment course and subsequent one-year observation also illustrate the complexity of assessing safety and durability in long-standing refractory disease. Treatment was discontinued when anemia worsened and required transfusion; later hematological and gastroenterological evaluations identified pre-existing disease- and iron-deficiency-related factors, leaving the contribution of baricitinib uncertain. Calcinosis progressed after treatment discontinuation. This case adds to the emerging real-world experience with JAK inhibition in dermatomyositis-associated calcinosis and supports prospective evaluation using standardized clinical, functional, imaging, and safety outcomes.

## Data Availability

The raw data supporting the conclusions of this article will be made available by the authors, without undue reservation.
